# Supplementary material for: The genome sequence of the common stiletto fly, Thereva nobilitata (Fabricius, 1775)
Source: Wellcome Open Res. 2025 Apr 8;10:183. [Version 1] doi: 10.12688/wellcomeopenres.23970.1 (PMC12485356; doi:10.12688/wellcomeopenres.23970.1)
Supplement: Peer review history

# Reviewer report: Reviewer response for version 1

**DOI:** 10.21956/wellcomeopenres.26446.r122145

**Published:** 2025-05-07

**Authors:** Anthony Bayega

**Affiliations:** 1 Canada's Michael Smith Genome Sciences Centre, Vancouver, British Columbia, Canada

McCullochet al. provide a high quality chromosomal-level genome assembly of *Thereva nobilitata*, a commendable effort. My comments follow below:

 1. In Figure 3, the label on the Y-axis which reads “ERR1…” could be changed to something more informative to help the reader make sense of this figure.

 2. It is commendable that the group has attempted to decontaminate the assembly of cobionts and other contaminants.

 Overall, the authors provide a high-quality genome and also assign 99.61% of it to chromosomes. Although much work remains to order the scaffolds, fully phase the contigs and scaffolds, and complete the gaps and also structurally and functionally annotate the genome, the current work will indeed be valuable to the whole community. I therefore recommend the indexing of this genome so that this resource becomes widely accessible to the scientific community.

Are sufficient details of methods and materials provided to allow replication by others?

Yes

Is the rationale for creating the dataset(s) clearly described?

Yes

Are the datasets clearly presented in a useable and accessible format?

Yes

Are the protocols appropriate and is the work technically sound?

Yes

Reviewer Expertise:

Genomics and human genetics

I confirm that I have read this submission and believe that I have an appropriate level of expertise to confirm that it is of an acceptable scientific standard.

# Reviewer report: Reviewer response for version 1

**DOI:** 10.21956/wellcomeopenres.26446.r122142

**Published:** 2025-04-29

**Authors:** Komal Kumar Bollepogu Raja

**Affiliations:** 1 Baylor College of Medicine, Houston, USA

McCulloch and Crowley present the genome assembly of an insect species *Thereva nobilitata*. The authors have provided ecological and morphological description of the species in the background. The methodology and the tools used for the genome assembly are standard and are presented well in the manuscript. This allows for reproducibility. The genome assembly appears to be high quality with BUSCO of 96.2% and k-mer completeness of 99.46%. 99x coverage of the genome and the 99.6% mapped to genome are impressive. Overall, the manuscript is well written and is a good resource for other researchers.

Are sufficient details of methods and materials provided to allow replication by others?

Yes

Is the rationale for creating the dataset(s) clearly described?

Yes

Are the datasets clearly presented in a useable and accessible format?

Yes

Are the protocols appropriate and is the work technically sound?

Yes

Reviewer Expertise:

Single cell genomics, genomics, bioinformatics and Neurobiology

I confirm that I have read this submission and believe that I have an appropriate level of expertise to confirm that it is of an acceptable scientific standard.
